# Supplementary material for: Significant contribution of subseafloor microparticles to the global manganese budget
Source: Nat Commun. 2019 Feb 6;10:400. doi: 10.1038/s41467-019-08347-2 (PMC6365551; doi:10.1038/s41467-019-08347-2)
Supplement: Supplementary file 1 — Supplementary Information [file 41467_2019_8347_MOESM1_ESM.pdf]

Supplementary Information

Significant contribution of subseafloor microparticles to the global manganese budget

Go-Ichiro Uramoto, Yuki Morono, Naotaka Tomioka, Shigeyuki Wakaki, Ryoichi Nakada, Rota Wagai, Kentaro Uesugi, Akihisa Takeuchi, Masato Hoshino, Yoshio Suzuki, Fumito Shiraishi, Satoshi Mitsunobu, Hiroki Suga, Yasuo Takeichi, Yoshio Takahashi & Fumio Inagaki

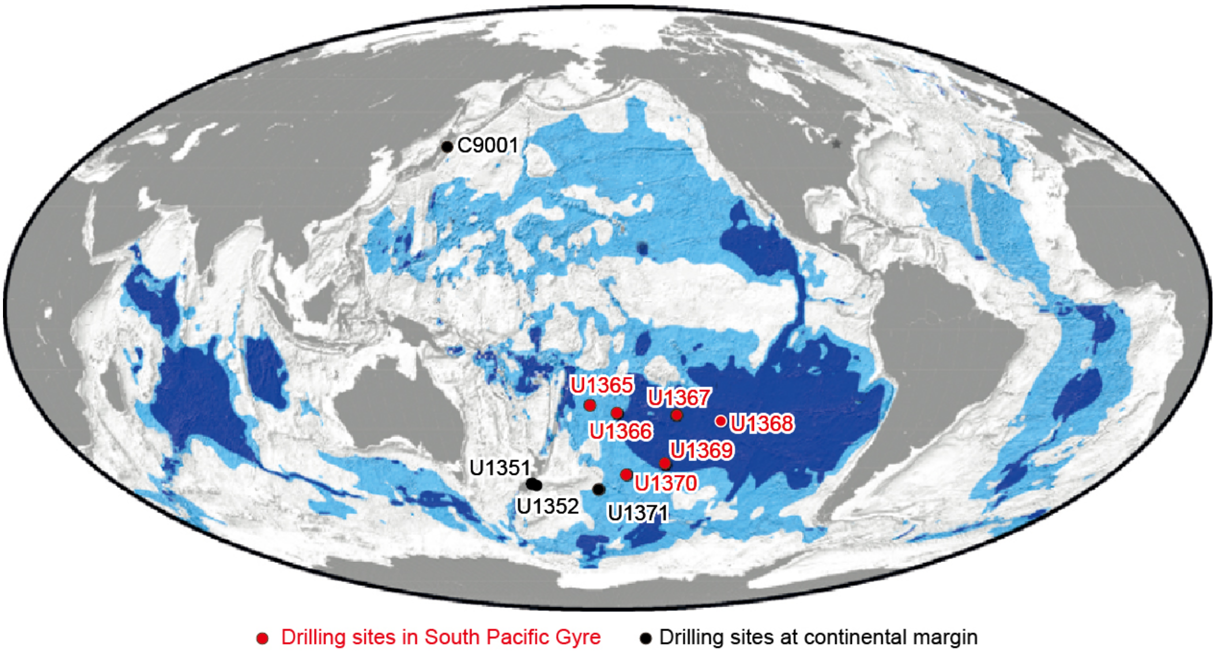

Supplementary Figure 1 | Locations of the sampling sites. Sites are plotted on a global map showing regions that may contain dissolved oxygen and aerobic activity through the entire sedimentary sequence[1]. Dark (light) blue indicates regions likely to feature maximum (minimum) dissolved oxygen and aerobic activity.

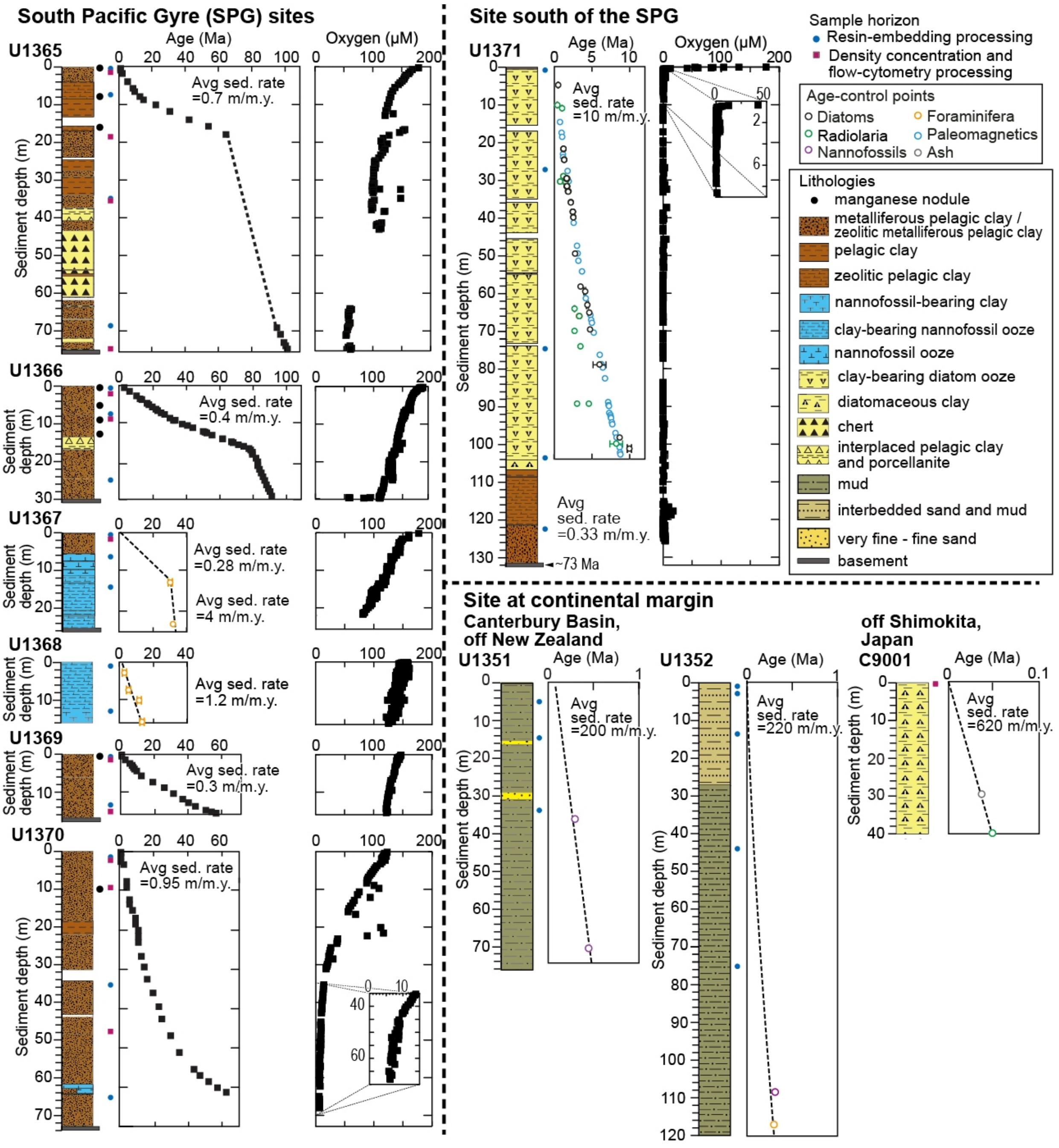

Supplementary Figure 2 | Lithology, age, and average sedimentation rates at sampling sites. At sites in the SPG, dissolved oxygen is also shown[1]. Lithology at Sites U1365–U1371 after D'Hondt et al.[2], at Sites U1365–U1352 after Fulthorpe et al.[3], and at Site C9001 after Aoike et al.[4]. Sediment ages at Sites U1365, U1366, U1369, and U1370 are from a cobalt-based age model[5], those at Sites U1367 and U1368 are based on foraminifers[2], those at Site U1371 are based on paleomagnetics[2] and siliceous microfossil[6,7], those at Sites U1351 and U1352 are based on calcareous and siliceous microfossils[3], and those at Site C9001 are based on siliceous microfossils and ash[4].

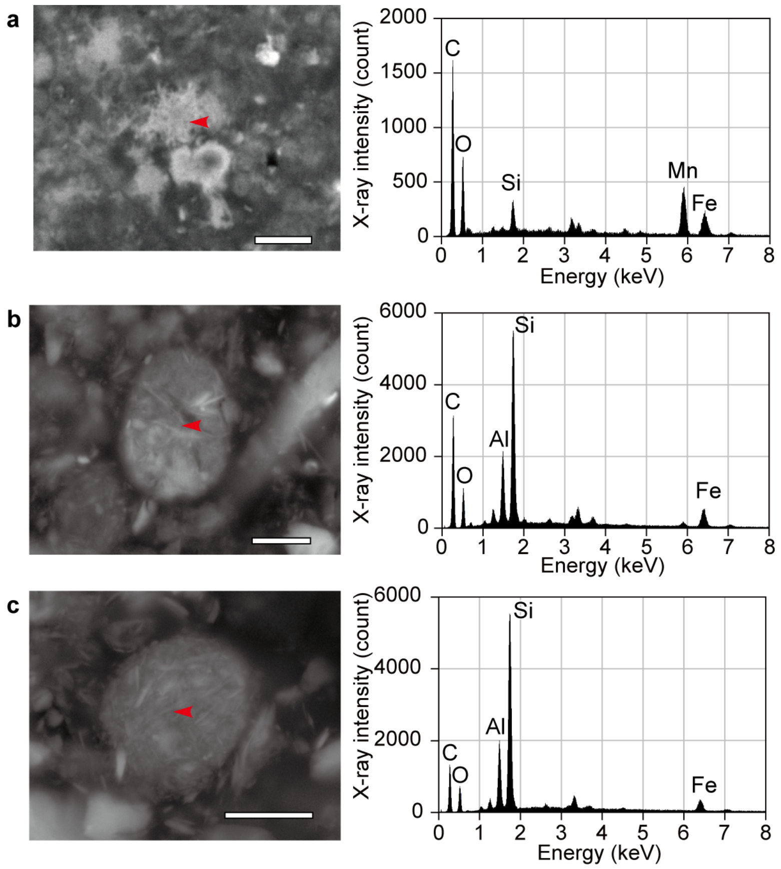

Supplementary Figure 3 | Representative SEM-EDS analysis results for microparticles in sediment samples. (**a**) Mn-microparticle in oxic pelagic clay (sample U1365A-24H-1 92/96). (**b**) Clay-microparticle in oxic pelagic clay (sample U1365A-2H-1 92/96). (**c**) Clay-microparticle in anoxic clay-bearing diatom ooze (sample U1371D-1H-1 67/71). Magenta arrows indicate analyzed points. Scale bars, 5 µm (**a**); 2 µm (**b**, **c**).

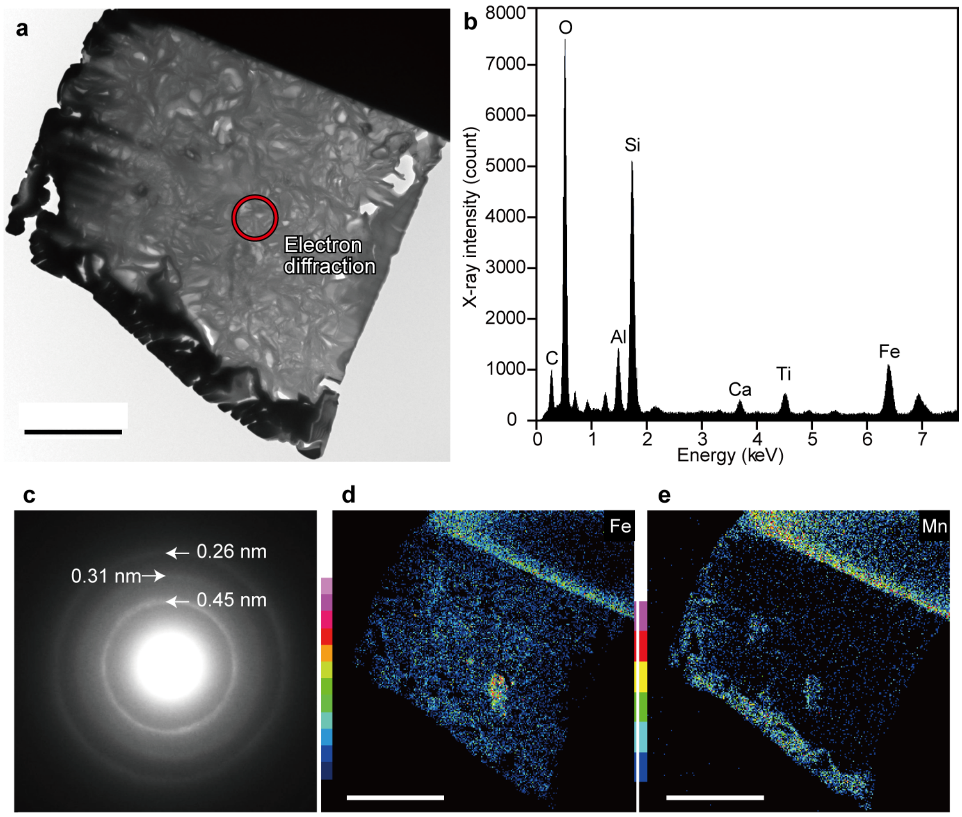

Supplementary Figure 4 | Representative TEM analysis results for clay-microparticles. (**a**) TEM image. (**b**) EDS spectrum. (**c**) Electron diffraction pattern. (**i–j**) Elemental maps for Fe and Mn, respectively. Based on the EDS spectrum and electron diffraction pattern, a nontronite-like mineral is the main mineral constituent. Scale bars, 2 μm (**a**, **d**, **e**).

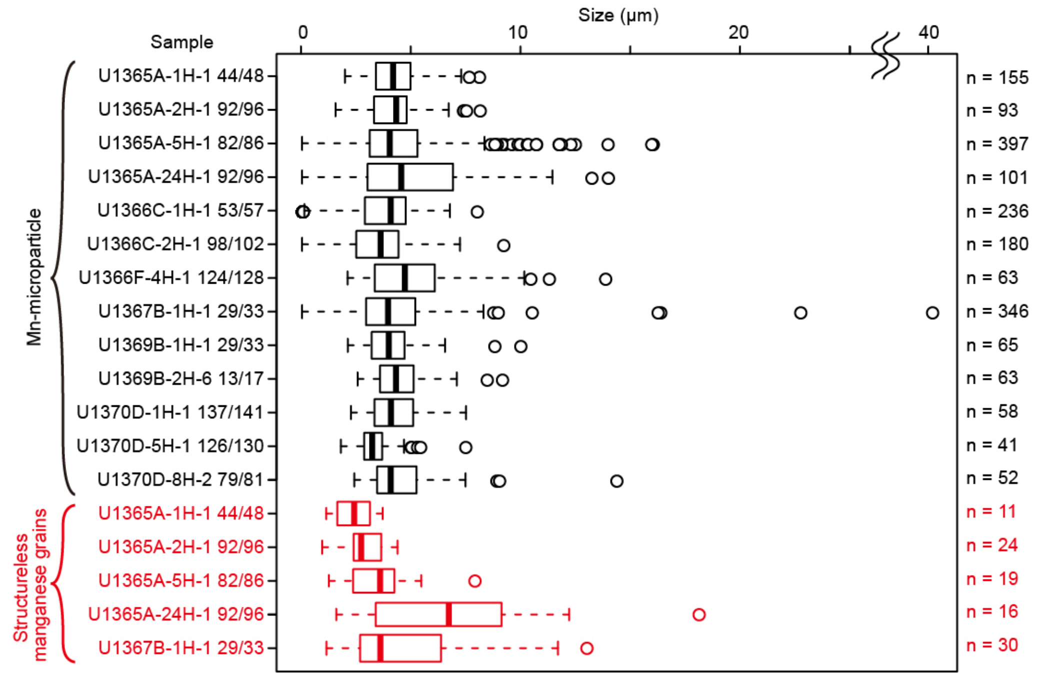

Supplementary Figure 5 | Box and whisker plots showing mean and associated errors in the size of Mn-microparticles and irregular-shaped manganese-concentrated minerals in the samples studied herein. The boundaries of the boxes represent the first and third quartiles, while the line within the box represents the second quartile (median). The whiskers represent the allowable data range (1.5 times the interquartile range). Circles represent outliers.

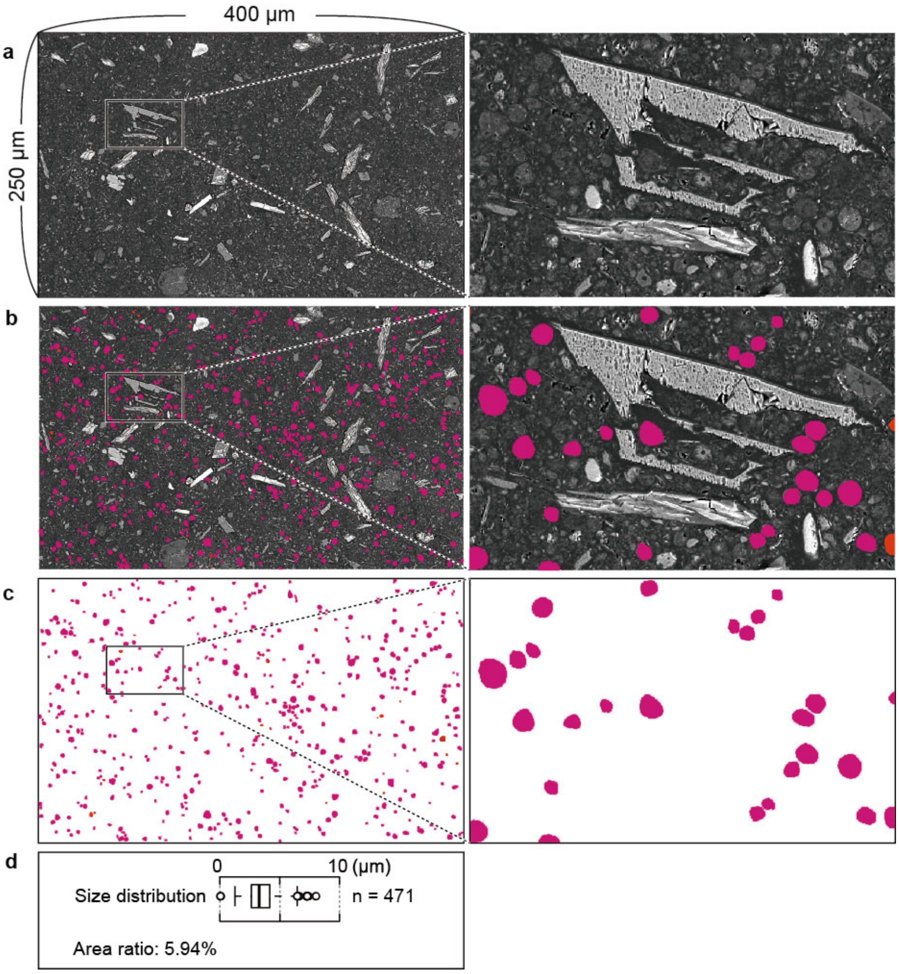

Supplementary Figure 6 | Image processing used to estimate the number of microparticles per unit volume of sample. (**a**) Back-scattered electron (BSE) image acquired via SEM (sample U1365C-2H-3 0/20). (**b**) Tracing microparticles (red) identified in the BSE image. (**c**) Extraction of microparticle areas. (**d**) Computed size distribution of the microparticle area ratio.

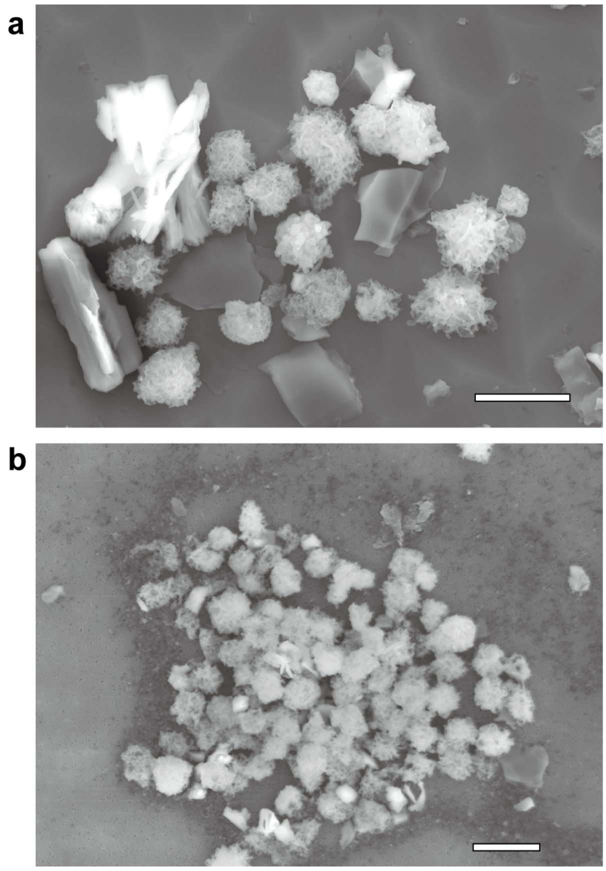

Supplementary Figure 7 | Representative SEM images showing the Mn-microparticle concentration and separation processing results. (**a**) Sample after density concentration processing. (**b**) Sample after combined processing by density concentration and flow cytometry/particle sorting. Sample: U1365C-1H-2 0/20. Scale bars, 10 µm (**a**, **b**).

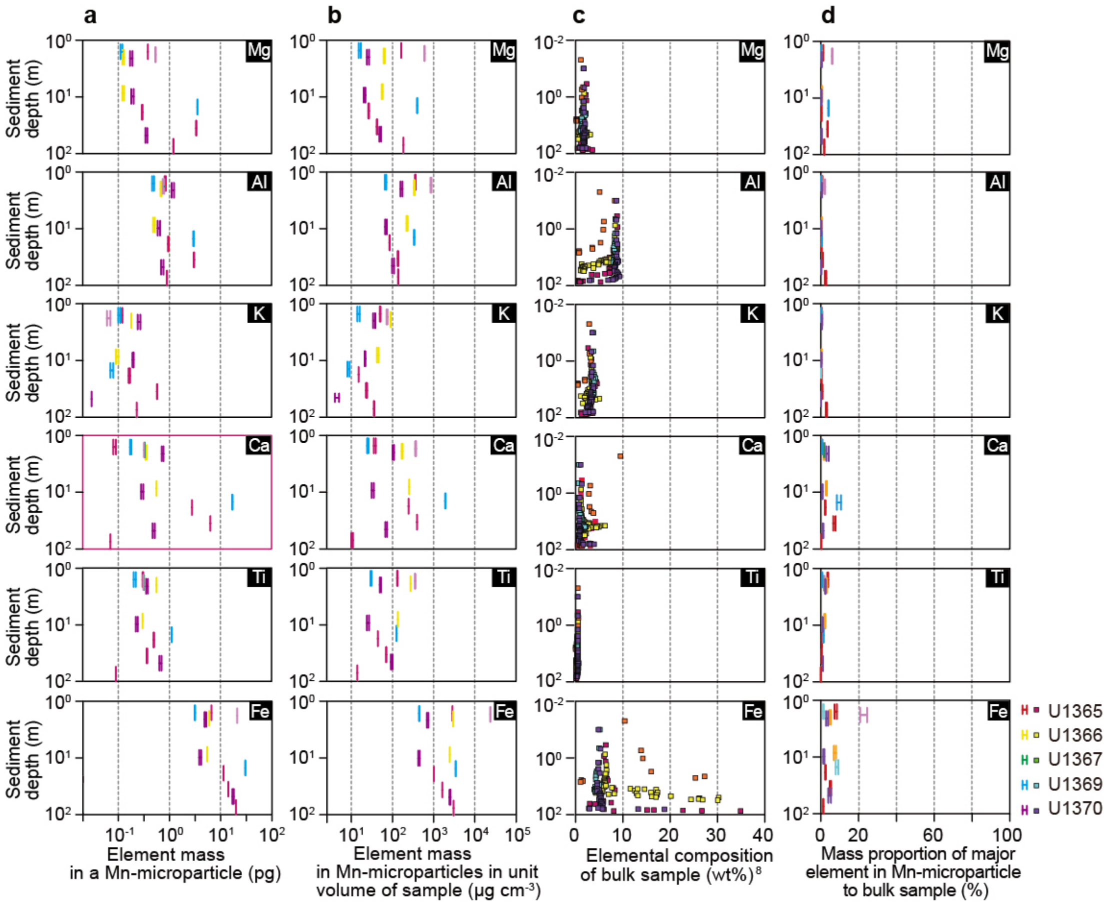

Supplementary Figure 8 | Changes with depth in mass and proportion of major elements (Mg, Al, K, Ca, Ti, and Fe) in Mn-microparticles and bulk sediment samples. (**a**) Average mass of major elements in a Mn-microparticle. (**b**) Average mass of major elements in Mn-microparticles in a unit volume of sample. (**c**) Major element composition in the bulk sample[8]. (**d**) Proportion of Mn-microparticle major element mass to bulk sample mass. Whiskers represent the minimum and maximum values based on the estimated minimum and maximum density (2.35 and 3.0 g cm^{-3} respectively) of the Mn-microparticle.

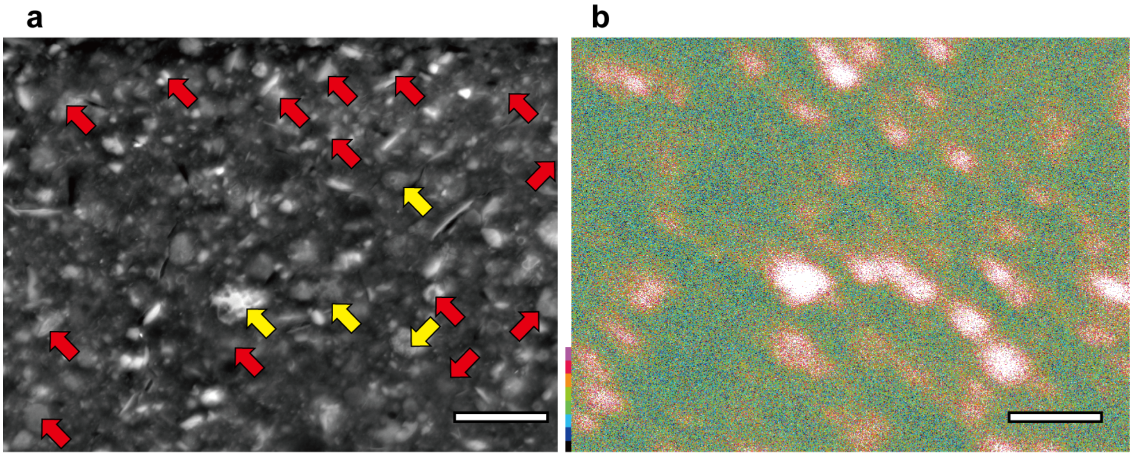

Supplementary Figure 9 | Representative distribution of manganese minerals in a resin-embedded sediment sample in this study. (**a**) BSE image acquired by SEM and (**b**) elemental map of manganese for sample U1365D-2H-1 92/96. Yellow arrows indicate Mn-microparticles. Red arrows indicate manganese-concentrated particles without concentric growth structure and fibrous structure. Scale bars, 10 μm (**a**, **b**).

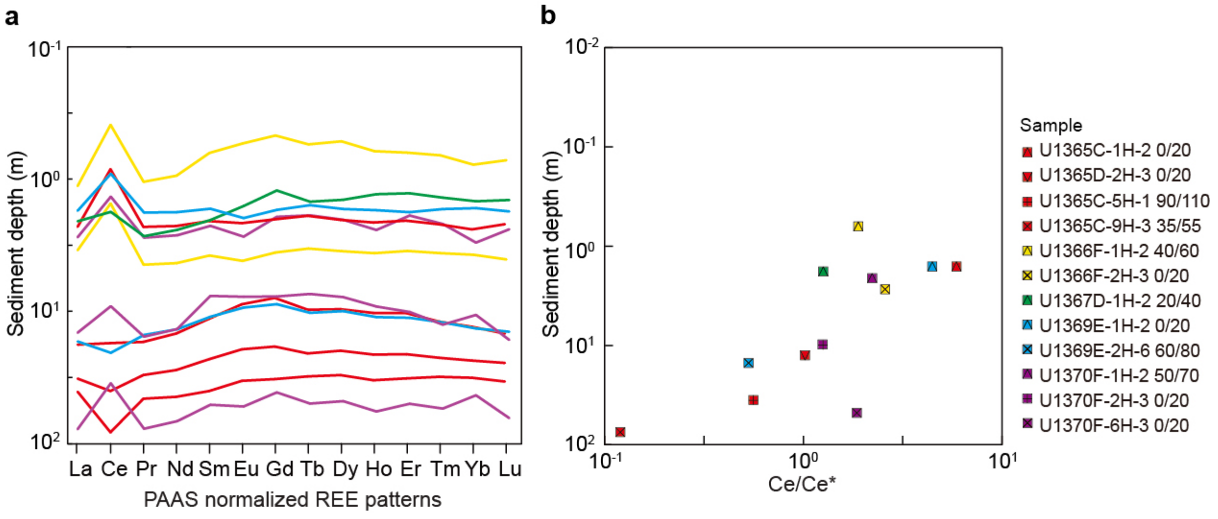

Supplementary Figure 10 | Content of REEs from enriched Mn-microparticles in oxic pelagic clays from the SPG. Changes in (**a**) PAAS-normalized REE patterns and (**b**) Ce/Ce* ratios with depth.

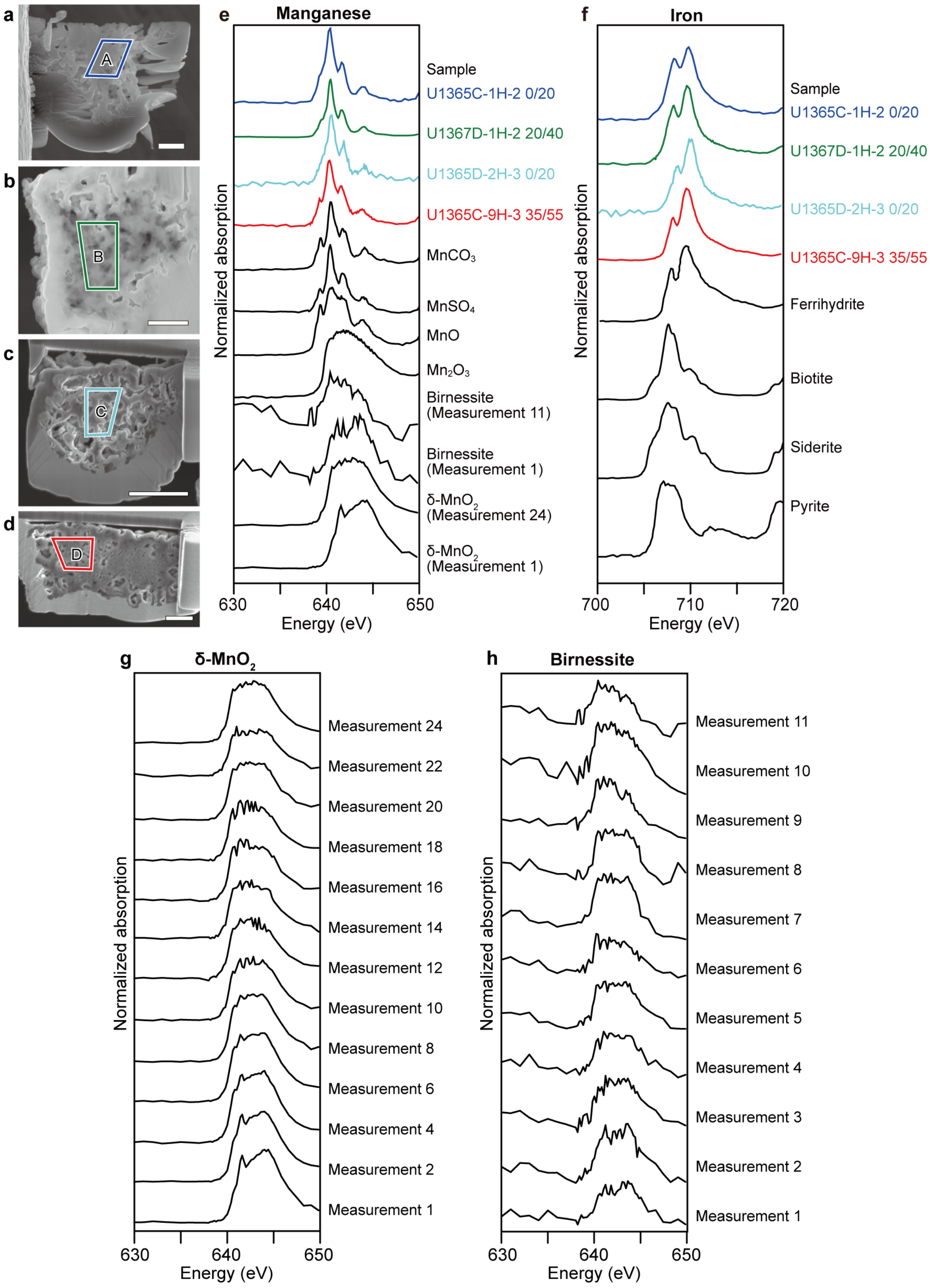

Supplementary Figure 11 | STXM results for the samples studied herein. (**a**–**f**) NEXAFS spectra of Mn and Fe for Mn-microparticles and standard samples. Cross-sectional SEM images of FIB-cut Mn-microparticles in STXM-analyzed samples (**a**) U1365C-1H-2 0/20, (**b**) U1367D-1H-2 20/40, (**c**) U1365D-2H-3 0/20, and (**d**) U1365C-9H-3 35/55. Solid squares indicate analyzed spot in the NEXAFS spectra. (**e**) NEXAFS spectra of the Mn L-edge. (**f**) NEXAFS spectra of the Fe L-edge. (**g**) Changes in the redox state of manganese in δ-MnO_2 during STXM measurements. (**h**) Changes in the redox state of manganese in birnessite during STXM measurements. Scale bars, 1μm (**a-d**).

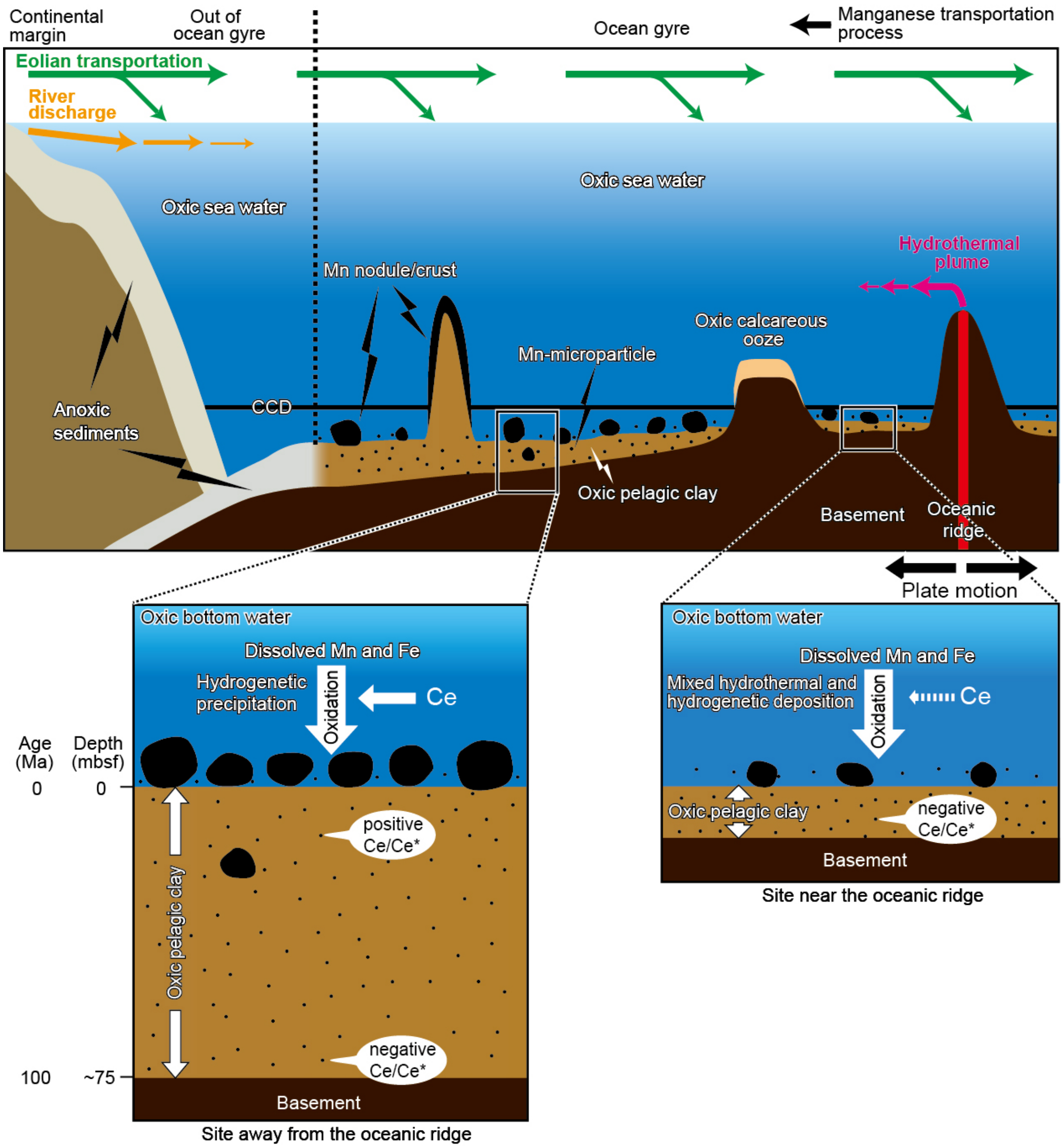

Supplementary Figure 12 | Schematic illustration showing the formation of Mn-microparticles in the bottom water and by burial into the oxic subseafloor environment over 100 m.y.

Supplementary Tables

Supplementary Table 1: Locations of core sampling sites

Site	Latitude	Longitude	Water depth (m)
Sites located in the South Pacific Gyre (SPG)[2]			
U1365	23°51.0493′S	165°38.6624′W	5695.6
U1366	26°03.0845′S	156°53.6700′W	5129.5
U1367	26°29.8966′S	137°56.3777′W	4288.9
U1368	27°55.0024′S	123°09.6679′S	3739.1
U1369	39°18.6178′S	139°48.0522′W	5275.2
U1370	41°51.1156′S	153°06.3812′W	5073.6
Site located south of the SPG[2]			
U1371	45°57.8394′S	163°11.0512′W	5311.09
Site located at the continental margin[3,4]			
U1351	44°53.0422′S	171°50.4065′E	121.7
U1352	44°56.2558′S	172°1.3630′E	343.6
C9001	41°10.5983′N	142°12.0328′E	1180

Supplementary Table 2: List of sediment samples

Sample	Sediment depth (m)	Lithology
Resin-embedding processing		
U1365A-1H-1 44/48	0.44–0.48	Metalliferous pelagic clay
U1365A-2H-1 92/96	7.12–7.16	Zeolitic pelagic clay
U1365A-5H-1 82/86	35.02–35.06	Zeolitic metalliferous pelagic clay
U1365A-24H-1 92/96	68.12–68.16	Metalliferous pelagic clay
U1366C-1H-1 53/57	0.53–0.57	Zeolitic metalliferous pelagic clay
U1366C-2H-1 98/102	7.12–7.16	Metalliferous pelagic clay
U1366F-4H-1 124/128	24.74–24.78	Metalliferous pelagic clay
U1367B-1H-1 29/33	0.29–0.33	Zeolitic metalliferous pelagic clay
U1367B-2H-1 76/80	6.26–6.30	Nannofossil ooze
U1367B-3H-1 64/68	14.14–14.18	Nannofossil ooze
U1368B-1H-1 84/86	0.84–0.86	Nannofossil ooze
U1368B-2H-6 53/57	13.03–13.07	Nannofossil-bearing clay
U1369B-1H-1 29/33	0.29–0.33	Zeolitic metalliferous pelagic clay
U1369B-2H-6 13/17	13.83–13.87	Zeolitic metalliferous pelagic clay
U1370D-1H-1 137/141	1.37–1.41	Zeolitic metalliferous pelagic clay
U1370D-5H-1 126/130	35.76–35.80	Zeolitic metalliferous pelagic clay
U1370D-8H-2 79/81	65.29–65.31	Metalliferous pelagic clay
U1371D-1H-1 67/71	0.67–0.71	Clay-bearing diatom ooze
U1371D-4H-1 107/111	27.47–27.51	Clay-bearing diatom ooze
U1371D-9H-1 93/97	74.83–74.87	Clay-bearing diatom ooze
U1371D-12H-1 97/101	103.37–103.41	Clay-bearing diatom ooze
U1371D-14H-1 83/87	122.23–122.27	Zeolitic metalliferous pelagic clay
U1351B-1H-4 22/24	4.72–4.74	Sandy silt
U1351B-2H-5 142/144	15.24–15.24	Silt
U1351B-6H-2 27/29	33.97–33.99	Sandy silt
U1352B-1H-1 113/115	1.13–1.15	Sandy silt
U1352B-1H-3 45/47	3.45–3.47	Silt
U1352B-2H-4 89/91	13.59–13.61	Sandy silt
U1352B-5H-6 106/108	43.98–44.00	Silty clay
U1352B-9H-1 26/28	75.83–75.85	Silt
KY11-E06 25/30	0.25–0.30	Diatomaceous clay
Density concentration and flow cytometry processing		
U1365C-1H-2 0/20	1.50–1.70	Metalliferous pelagic clay
U1365D-2H-3 0/20	18.50–18.70	Metalliferous pelagic clay
U1365C-5H-1 90/110	35.40–35.60	Metalliferous pelagic clay
U1365C-9H-3 35/55	74.35–74.55	Metalliferous pelagic clay
U1366F-1H-2 40/60	1.90–2.10	Zeolitic metalliferous pelagic clay
U1366F-2H-3 0/20	8.50–8.70	Metalliferous pelagic clay
U1367D-1H-2 20/40	1.70–1.90	Zeolitic metalliferous pelagic clay
U1369E-1H-2 0/20	1.50–1.70	Zeolitic metalliferous pelagic clay
U1369E-2H-6 60/80	14.90–15.10	Zeolitic metalliferous pelagic clay
U1370F-1H-2 50/70	2.00–2.20	Zeolitic metalliferous pelagic clay
U1370F-2H-3 0/20	9.70–9.90	Zeolitic metalliferous pelagic clay
U1370F-6H-3 0/20	47.70–47.90	Metalliferous pelagic clay
KY11-E06 25/30	0.25–0.30	Diatomaceous clay

Supplementary Table 3: ICP-MS analysis results for enriched Mn-microparticles

		1365D-1H-2 0/20	1365D-2H-3 0/20	1365C-5H-1 90/110	1365C-9H-1 35/55	1366F-1H-2 40/60	1366F-2H-3 0/20	1367D-1H-2 20/40	1369E-1H-3 0/20	1369E-2H-6 60/80	1370F-1H-2 50/70	1370F-2H-3 0/20	1370F-6H-3 0/20	NaCl solution	SPT solution
Mg	[ng]	3.9×10^{1}	4.9×10^{1}	4.6×10^{2}	1.4×10^{2}	3.3×10^{1}	2.7×10^{1}	6.2×10^{1}	5.4×10^{1}	4.6×10^{2}	1.3×10^{2}	8.5×10^{1}	1.3×10^{2}		1×10^{-1}
Al	[ng]	1.5×10^{2}	1.5×10^{2}	4.6×10^{2}	1.2×10^{2}	1.2×10^{2}	1.2×10^{2}	1.6×10^{2}	1.9×10^{2}	5.2×10^{2}	4.8×10^{2}	5.1×10^{2}	3.0×10^{2}	4.0×10^{-1}	
K	[ng]	2.6×10^{1}	2.9×10^{1}	7.4×10^{1}	3.0×10^{1}	2.5×10^{1}	3.5×10^{1}	1.3×10^{1}	6.9×10^{1}	3.9×10^{1}	2.1×10^{2}	1.7×10^{2}	1.2×10^{2}		
Ca	[ng]	7.0×10^{1}	3.2×10^{2}	7.6×10^{2}	3.0×10^{1}	7.6×10^{1}	5.6×10^{1}	6.5×10^{1}	9.3×10^{1}	2.5×10^{3}	1.1×10^{2}	2.9×10^{2}	8.6×10^{1}	3.3×10^{-1}	1.1×10^{0}
Ti	[ng]	5.3×10^{1}	6.2×10^{1}	6.9×10^{1}	1.2×10^{1}	5.7×10^{1}	2.9×10^{1}	4.3×10^{1}	4.2×10^{1}	1.4×10^{2}	5.7×10^{1}	7.5×10^{1}	1.1×10^{2}		
Mn	[ng]	3.7×10^{2}	1.5×10^{3}	6.3×10^{3}	5.2×10^{2}	7.3×10^{2}	7.7×10^{2}	7.8×10^{2}	7.5×10^{2}	5.9×10^{3}	3.5×10^{2}	2.6×10^{2}	4.4×10^{2}	3.0×10^{-2}	8×10^{-2}
Fe	[ng]	6.7×10^{2}	1.3×10^{3}	1.7×10^{3}	2.3×10^{3}	6.1×10^{2}	5.5×10^{2}	2.3×10^{3}	3.2×10^{2}	3.7×10^{3}	5.0×10^{2}	2.7×10^{2}	7.6×10^{2}	1.9×10^{-1}	
Co	[ng]	3.8×10^{0}	1.8×10^{1}	4.3×10^{1}	6.8×10^{-1}	1.1×10^{1}	1.5×10^{1}	5.2×10^{0}	1.0×10^{1}	6.1×10^{1}	4.7×10^{0}	3.4×10^{0}	4.2×10^{0}		
Ni	[ng]	1.6×10^{0}	2.7×10^{1}	2.5×10^{2}	3.7×10^{0}	1.0×10^{1}	1.3×10^{1}	1.5×10^{1}	1.8×10^{1}	3.7×10^{2}	7.7×10^{0}	1.2×10^{1}	1.6×10^{1}	1.9×10^{-1}	
Cu	[ng]	4.2×10^{-1}	7.3×10^{0}	1.2×10^{2}	6.2×10^{0}	6.8×10^{0}	2.7×10^{0}	1.4×10^{1}	8.5×10^{0}	1.1×10^{2}	2.8×10^{0}	1.6×10^{0}	5.2×10^{0}	1.3×10^{-1}	7×10^{-2}
Zn	[ng]	4.8×10^{-1}	5.0×10^{0}	3.1×10^{1}	2.2×10^{0}	2.2×10^{0}	1.7×10^{0}	6.1×10^{0}	1.5×10^{0}	3.6×10^{1}	1.5×10^{0}	1.1×10^{0}	3.3×10^{0}	1.2×10^{-2}	2×10^{-2}
Li	[pg]	6.3×10^{1}	1.8×10^{2}	2.5×10^{3}	2.1×10^{2}	8.8×10^{1}	1.4×10^{2}	5×10^{1}	1.8×10^{2}	1.2×10^{3}	5.8×10^{2}	3.5×10^{2}	6.0×10^{2}		
Sc	[pg]	5.1×10^{1}	5.0×10^{2}	4.8×10^{2}	7.7×10^{1}	1.1×10^{2}	1.2×10^{2}	1.4×10^{2}	8.0×10^{1}	1.8×10^{3}	1.0×10^{2}	1.5×10^{2}	1.2×10^{2}		
V	[pg]	7.5×10^{2}	3.3×10^{3}	6.0×10^{3}	3×10^{3}	1.2×10^{3}	8.2×10^{2}	5.3×10^{3}	9.2×10^{2}	1.1×10^{4}	1.2×10^{3}	1.4×10^{3}	1.9×10^{3}		
Cr	[pg]	6.7×10^{2}	1.1×10^{3}	1.2×10^{3}	1.4×10^{2}	5.2×10^{2}	2.2×10^{2}	2.0×10^{2}	5.3×10^{2}	3.7×10^{3}	8.9×10^{2}	5.4×10^{2}	9.2×10^{2}	1.7×10^{-2}	
Ga	[pg]	9×10^{1}	2.6×10^{2}	1.1×10^{3}	1.1×10^{2}	1.6×10^{2}	1.2×10^{2}	1.5×10^{2}	1.9×10^{2}	1.9×10^{3}	2.0×10^{2}	2.1×10^{2}	2.2×10^{2}		
As	[pg]	2.5×10^{2}	1.1×10^{3}	1.2×10^{3}	4.7×10^{2}	5.9×10^{2}	3.2×10^{2}	2.3×10^{3}	2.3×10^{2}	3.5×10^{3}	1.3×10^{2}	7.9×10^{1}	2.1×10^{2}		
Rb	[pg]	8.6×10^{1}	1.4×10^{2}	2.3×10^{2}	1.5×10^{2}	1.5×10^{2}	1.8×10^{2}	9.2×10^{1}	3.9×10^{2}	2.7×10^{2}	1.3×10^{3}	8.6×10^{2}	8.8×10^{2}	2.7×10^{1}	1×10^{1}
Sr	[pg]	5.2×10^{2}	3.8×10^{3}	9.3×10^{3}	3.0×10^{3}	9.0×10^{2}	1.1×10^{3}	2.2×10^{3}	1.5×10^{3}	1.4×10^{4}	1.3×10^{3}	3.7×10^{3}	9.8×10^{2}		3×10^{0}
Y	[pg]	1.1×10^{2}	6.3×10^{3}	1.5×10^{4}	6.1×10^{2}	1.0×10^{3}	3.7×10^{2}	1.4×10^{3}	1.6×10^{2}	2.8×10^{4}	2.0×10^{2}	3.5×10^{2}	5.5×10^{2}	9.8×10^{0}	
Zr	[pg]	5.1×10^{2}	3.8×10^{3}	4.5×10^{3}	9.0×10^{2}	1.3×10^{3}	1.3×10^{3}	3.8×10^{3}	4.8×10^{2}	1.3×10^{4}	4.5×10^{2}	2.9×10^{2}	1.1×10^{3}	1.4×10^{0}	3×10^{0}
Nb	[pg]	9.8×10^{1}	4.1×10^{2}	2.5×10^{2}	2.4×10^{1}	2.2×10^{2}	1.3×10^{2}	3.1×10^{2}	1.4×10^{2}	7.5×10^{2}	1.3×10^{2}	1.7×10^{2}	2.5×10^{2}		
Mo	[pg]	4.1×10^{2}	1.0×10^{3}	3.0×10^{3}	7.6×10^{1}	5.3×10^{2}	3.9×10^{2}	6.2×10^{2}	1.8×10^{3}	4.3×10^{3}	9.5×10^{2}	6.8×10^{2}	4.5×10^{2}		
Cd	[pg]	8×10^{0}	1.5×10^{1}	8.9×10^{1}		1.5×10^{1}	1×10^{1}	1×10^{1}	1.5×10^{1}	8.4×10^{1}					
Cs	[pg]	8×10^{0}	9.1×10^{0}	9.7×10^{0}		8.6×10^{0}	1.1×10^{1}	5×10^{0}	3.2×10^{1}	7×10^{0}	9.6×10^{1}	4.7×10^{1}	8.1×10^{1}		
Ba	[pg]	2.1×10^{3}	1.2×10^{4}	9.5×10^{4}	2.9×10^{4}	7.2×10^{3}	4.1×10^{3}	1.1×10^{4}	7.6×10^{4}	5.2×10^{5}	3.9×10^{4}	1.1×10^{4}	8.5×10^{3}		2×10^{1}
La	[pg]	1.2×10^{2}	4.6×10^{3}	1.1×10^{4}	8.7×10^{2}	9.6×10^{2}	5.3×10^{2}	1.3×10^{3}	2.4×10^{2}	1.8×10^{4}	2.4×10^{2}	4.0×10^{2}	5.4×10^{2}	2.2×10^{0}	1×10^{0}
Ce	[pg]	1.5×10^{3}	9.8×10^{3}	1.3×10^{4}	2.1×10^{2}	3.9×10^{3}	2.4×10^{3}	3.1×10^{3}	2.0×10^{3}	2.1×10^{4}	1.1×10^{3}	1.0×10^{3}	2.1×10^{3}	9.2×10^{0}	6×10^{0}
Pr	[pg]	2.8×10^{1}	1.1×10^{3}	2.8×10^{3}	1.7×10^{2}	2.4×10^{2}	7.7×10^{1}	2.3×10^{2}	4.6×10^{1}	5.2×10^{3}	5.5×10^{1}	9.0×10^{1}	1.2×10^{2}	3.9×10^{-1}	5×10^{-1}
Nd	[pg]	1.2×10^{2}	5.0×10^{3}	1.3×10^{4}	6.8×10^{2}	9.9×10^{2}	3.1×10^{2}	1.0×10^{3}	1.9×10^{2}	2.3×10^{4}	2.3×10^{2}	3.7×10^{2}	5.5×10^{2}	5.0×10^{0}	
Sm	[pg]	2.6×10^{1}	1.0×10^{3}	2.6×10^{3}	1.3×10^{2}	2.1×10^{2}	6.6×10^{1}	1.9×10^{2}	4.0×10^{1}	5.0×10^{3}	4.7×10^{1}	7.7×10^{1}	1.1×10^{2}	4.5×10^{0}	
Eu	[pg]	4.5×10^{0}	2.3×10^{2}	6.2×10^{2}	3.0×10^{1}	4.5×10^{1}	1.1×10^{1}	4.6×10^{1}	2.5×10^{0}	1.1×10^{3}	6.9×10^{0}	1.5×10^{1}	2.2×10^{1}	2.2×10^{0}	1×10^{0}
Gd	[pg]	2.4×10^{1}	1.1×10^{3}	2.8×10^{3}	1.3×10^{2}	2.1×10^{2}	6.1×10^{1}	2.3×10^{2}	3.1×10^{1}	5.2×10^{3}	4.7×10^{1}	6.4×10^{1}	1.1×10^{2}	1.1×10^{0}	
Tb	[pg]	4.8×10^{0}	1.6×10^{2}	4.1×10^{2}	2.3×10^{1}	3.2×10^{1}	1.1×10^{1}	3.4×10^{1}	$7.1\times1^{0}0$	7.5×10^{2}	$8.0\times1^{0}0$	1.1×10^{1}	1.6×10^{1}	$5.8\times1^{0}0$	1×10^{0}
Dy	[pg]	2.4×10^{1}	9.6×10^{2}	2.6×10^{3}	1.4×10^{2}	2.0×10^{2}	6.3×10^{1}	2.1×10^{2}	3.3×10^{1}	4.7×10^{3}	4.5×10^{1}	6.4×10^{1}	1.0×10^{2}	4.5×10^{-1}	
Ho	[pg]	4.3×10^{0}	1.9×10^{2}	5.2×10^{2}	2.8×10^{1}	3.8×10^{1}	1.3×10^{1}	4.8×10^{1}	6.4×10^{0}	8.9×10^{2}	7.6×10^{0}	1.3×10^{1}	1.8×10^{1}		
Er	[pg]	1.4×10^{1}	5.6×10^{2}	1.5×10^{3}	8.3×10^{1}	1.1×10^{2}	3.9×10^{1}	1.4×10^{2}	1.5×10^{1}	2.5×10^{3}	2.9×10^{1}	3.5×10^{1}	5.9×10^{1}		
Tm	[pg]	1.6×10^{0}	7.1×10^{1}	2.0×10^{2}	1.2×10^{1}	1.5×10^{1}	5.2×10^{0}	1.9×10^{1}	2.9×10^{0}	3.3×10^{2}	3.5×10^{0}	4.5×10^{0}	7.9×10^{0}		
Yb	[pg]	7×10^{0}	4.6×10^{2}	1.3×10^{3}	8.3×10^{1}	9.4×10^{1}	3.4×10^{1}	1.3×10^{2}	2.1×10^{1}	2.0×10^{3}	1.5×10^{1}	3.4×10^{1}	6.5×10^{1}		
Lu	[pg]	1.7×10^{0}	6.3×10^{1}	1.9×10^{2}	1.2×10^{1}	1.5×10^{1}	4.6×10^{0}	2.0×10^{1}	2.5×10^{0}	2.8×10^{2}	3.4×10^{0}	4.3×10^{0}	7.3×10^{0}		
Hf	[pg]	8.7×10^{0}	6.9×10^{1}	7.5×10^{1}	9.2×10^{0}	1.9×10^{1}	1.3×10^{1}	8.2×10^{1}	9.1×10^{0}	2.4×10^{2}	1.1×10^{1}	8.5×10^{0}	2.0×10^{1}	1.9×10^{0}	
Ta	[pg]	3.8×10^{0}	1.5×10^{1}	1.0×10^{1}	1×10^{0}	7.8×10^{0}	4.5×10^{0}	7.2×10^{0}	8.9×10^{0}	1.2×10^{1}	9.9×10^{0}	1.3×10^{1}	1.3×10^{1}		
Tl	[pg]	2.9×10^{1}	1.4×10^{2}	1.2×10^{3}		8.6×10^{1}	1.1×10^{2}	1.4×10^{2}	4.9×10^{1}	6.7×10^{2}	1.6×10^{1}	2.2×10^{1}	5.2×10^{1}		
Pb	[pg]	2.7×10^{3}	3.5×10^{3}	7.3×10^{3}	6.9×10^{2}	3.4×10^{3}	2.0×10^{3}	3.3×10^{3}	2.7×10^{3}	1.1×10^{4}	1.4×10^{3}	7.9×10^{2}	1.8×10^{3}	1.0×10^{1}	1.0×10^{0}
Th	[pg]	8.7×10^{1}	3.4×10^{2}	2.2×10^{2}	6×10^{0}	1.5×10^{2}	2.4×10^{1}	2.8×10^{1}	9.0×10^{1}	9.4×10^{2}	1.6×10^{2}	1.1×10^{2}	8.3×10^{1}	1.2×10^{0}	
U	[pg]	6.3×10^{0}	1.1×10^{2}	1.1×10^{2}	1.5×10^{1}	3.0×10^{1}	3.8×10^{1}	5.7×10^{1}	2.4×10^{1}	5.6×10^{2}	1.8×10^{1}	3.5×10^{1}	3.5×10^{1}		

Supplementary References

1. D'Hondt, S. *et al.* Presence of oxygen and aerobic communities from sea floor to basement in deep-sea sediments. *Nat. Geosci.* **8,** 299–304 (2015).

2. D'Hondt, S., Inagaki, F., Alvarez Zarikian, C. A. & the Expedition 329 Scientists. *Proc. IODP, 329*. (Integrated Ocean Drilling Program Management International, Inc., for the Integrated Ocean Drilling Program, 2011). doi:10.2204/iodp.proc.329.2011

3. Fulthorpe, C.S., Hoyanagi, K., Blum, P. & the Expedition 317 Scientists. *Proc. IODP, 317*. (Integrated Ocean Drilling Program Management International, Inc., for the Integrated Ocean Drilling Program, 2011). doi:10.2204/iodp.proc.317.2011

4. Aoike, K. *et al.* Paleoceanographic history of offshore Shimokita Peninsula for the past 800,000 years based on primary analyses on cores recovered by D/V CHIKYU during the shakedown cruises. *Fossils.* **87**, 65–81 (2010).

5. Dunlea, A. G. *et al.* Cobalt-based age models of pelagic clay in the South Pacific Gyre. *Geochemistry, Geophys. Geosystems* **16,** 2694–2710 (2015).

6. Suto, I. & Uramoto, G.-I. in D'Hondt, S., Inagaki, F., Alvarez Zarikian, C.A., and the Expedition 329 Scientists. *Proc. IODP, 329*, doi:10.2204/iodp.proc.329.203.2015

7. Cortese, G. & Alvarez Zarikian, C. A. in D'Hondt, S., Inagaki, F., Alvarez Zarikian, C.A., and the Expedition 329 Scientists. *Proc. IODP, 329*, doi:10.2204/iodp.proc.329.202.2015

8. Dunlea, A. G. *et al.* Dust, volcanic ash, and the evolution of the South Pacific Gyre through the Cenozoic. *Paleoceanography* **30,** 1078–1099 (2015).
