# Supplementary material for: Significant contribution of subseafloor microparticles to the global manganese budget
Source: Nat Commun. 2019 Feb 6;10:400. doi: 10.1038/s41467-019-08347-2 (PMC6365551; doi:10.1038/s41467-019-08347-2)
Supplement: Supplementary file 3 — Description of Additional Supplementary Files [file 41467_2019_8347_MOESM3_ESM.pdf]

Description of Additional Supplementary Files

File Name: Supplementary Movie 1
Description: Cross-sectional X-ray micro-computed tomography image of a Mn-microparticle.
Sample: U1365C-1H-2 0/20.

File Name: Supplementary Movie 2
Description: Three-dimensional X-ray micro-computed tomography image of a Mn-microparticle.
Sample: U1365C-1H-2 0/20.
